# Preformulation Studies and Bioavailability Enhancement of Curcumin with a ‘Two in One’ PEG-β-Cyclodextrin Polymer

**DOI:** 10.3390/pharmaceutics13101710

**Published:** 2021-10-16

**Authors:** Ádám Haimhoffer, Eleftheria Dossi, Monika Béresová, Ildikó Bácskay, Judit Váradi, Ashfaq Afsar, Ágnes Rusznyák, Gábor Vasvári, Ferenc Fenyvesi

**Affiliations:** 1 Department of Pharmaceutical Technology, Faculty of Pharmacy, University of Debrecen, Nagyerdei St. 98, H-4032 Debrecen, Hungary; haimhoffer.adam@pharm.unideb.hu (Á.H.); bacskay.ildiko@pharm.unideb.hu (I.B.); varadi.judit@pharm.unideb.hu (J.V.); rusznyak.agnes@euipar.unideb.hu (Á.R.); vasvari.gabor@pharm.unideb.hu (G.V.); 2 Doctoral School of Pharmaceutical Sciences, University of Debrecen, H-4032 Debrecen, Hungary; 3 Institute of Healthcare Industry, University of Debrecen, Nagyerdei St. 98, H-4032 Debrecen, Hungary; 4 Cranfield Defence and Security, Cranfield University, Shrivenham, Swindon SN6 8LA, UK; e.dossi@cranfield.ac.uk (E.D.); Ashfaq.Afsar@cranfield.ac.uk (A.A.); 5 Department of Medical Imaging, University of Debrecen, Nagyerdei krt. 94, H-4032 Debrecen, Hungary; beres.monika@med.unideb.hu

**Keywords:** curcumin, PEG-cyclodextrin polymer, complexation, bioavailability, NMR

## Abstract

Drug delivery systems are used to improve the biopharmaceutical properties of curcumin. Our aim was to investigate the effect of a water-soluble ‘two in one’ polymer containing covalently bonded PEG and βCD moieties (βCPCD) on the solubility and bioavailability of curcumin and compare it to a polymeric β-cyclodextrin (βCDP) cross-linked with epichlorohydrin. Phase-solubility and dynamic light scattering (DLS) experiments showed that the solubility of curcumin increased significantly in 10 *m*/*m* % βCPCD and βCDP solutions, but βCPCD–curcumin particles had higher hydrodynamic volume. The formation of the βCPCD–curcumin complex in solution and sedimented phase was confirmed by NMR spectroscopy. Biocompatibility and permeability experiments were performed on Caco-2 cells. Polymers did not show cytotoxicity up to 10 *m*/*m* % and βCPCD significantly increased the permeability of curcumin. DLS measurements revealed that among the interaction of polymers with mucin, βCPCD formed bigger aggregates compared to βCDP. Curcumin complexes were lyophilized into capsules and structurally characterized by micro-CT spectroscopy. Drug release was tested in a pH 1.2 medium. Lyophilized complexes had a solid porous matrix and both βCPCD and βCDP showed rapid drug release. βCPCD provides an opportunity to create a swellable, mucoadhesive matrix system for oral drug delivery.

## 1. Introduction

Covalently linked cyclodextrin-containing macromolecular drug carrier systems have been investigated since the 1980s [1]. Several types of these macromolecular systems can be distinguished according to their structure such as linear polymers containing pendant cyclodextrin (CD) moieties [2], cross-linked cyclodextrin polymers [3,4] or polyrotaxanes [5,6,7]. Epichlorohydrin is a widely used cross-linker for the synthesis of cyclodextrin polymers [8], that are used for solubilization and stabilization of water-insoluble chemical substances [9,10]. Various drug molecules and bioactive agents such as doxorubicin and artemisinin [11], glipizide [12], triclosan [13], naproxen [14] and paclitaxel [15] were complexed by these water-soluble cyclodextrin polymers for drug delivery studies. By manipulating the cross-linking reaction conditions, such as the addition rate of cross-linker, reaction temperature and time, soluble or insoluble cyclodextrin polymers in water and/or other organic solvents are produced [3,16,17,18,19].

Cyclodextrin and polymer binary systems are also used to improve the solubilization of poorly soluble drugs [20]. To achieve higher degrees of drug solubilization, the synergistic effect of an active pharmaceutical ingredient (API), a cyclodextrin and a water-soluble polymer in solution was reported [21]. The formed ternary complex gives an alternative way for solubilization, especially when a high amount of CD is needed for complexation. The interaction of water-soluble polymers with drug molecules may occur by means of ion–ion, ion–dipole and dipole–dipole electrostatic bonds, van der Waals force, or 3-center or 2-electron bonds [22]. The water solubility and absence of biological activity are the most important requirements to choose polymers for complexes [22]. The most frequently used polymers for this purpose may be classified as natural, semi-synthetic and synthetic polymers [22]. The semi-synthetic polymers are carboxymethyl cellulose [21,23,24] and hydroxypropyl methylcellulose [25,26]. In several studies, povidone, polyvinyl alcohol and polyethylene glycol (PEG) promoted the complexation of drugs as a synthetic polymer suitable for ternary complex formation [27,28,29,30]. PEG is widely used as excipient or adjuvant in a variety of pharmaceutical formulations [31]. Nevertheless, CDs can be covalently linked to form polymers. CD polymers containing PEG soft units have been used to synthesize drug carrier gels for pharmaceutical applications [32,33,34,35]; however, in our knowledge, a water-soluble polyethylene glycol cross-linked β-CD polymer has never been tested as a drug carrier. Recently, semi-synthetic polymeric systems based on beta and gamma cyclodextrins were produced in the U.K. [16,17,18,19] from renewable resources and by ‘green chemistry’ processes. The green chemistry concept applies throughout the entire life cycle of a chemical product, from its design and manufacturing to its use and final disposal [36] and relates to the chemicals, process and products. The properties of crystalline β-cyclodextrin have been modified by cross-linking in water with a family of non-toxic diepoxides having variable length of polyethylene glycol spacers. Two different polymeric systems with large hydrodynamic volumes were obtained: soluble cross-linked polymers with defined chemistry and chemical-physical properties and insoluble hydrogels with good physical integrity and important swelling power up to 200% [37,38]. The new polymeric cyclodextrin-based systems are promising passive and/or active ingredients for a variety of biomedical applications. Our concept was to test the water-soluble polyethylene glycol cross-linked β-CD polymer as a ‘two in one’ polymeric system, which offers a combination of β-CD and PEG properties. Curcumin was chosen as a model drug, which can be solubilized by cyclodextrins [39], cyclodextrin polymers [39] and PEG 400 [40].

The aim of this work was to study the complexation properties of a water-soluble polyethylene glycol cross-linked β-CD polymer (βCPCD) using curcumin as a model drug molecule and compare these properties to a water-soluble cross-linked with an epichlorohydrin β-CD polymer (βCDP)/curcumin system. We characterized the interaction of βCPCD with mucin and determined curcumin solubilization. We also aimed to obtain information about the role of the PEG spacer in the molecular interactions and reveal the complexation properties and permeability enhancement of βCPCD. Finally, a hard gel capsule formulation was prepared, and the dissolution profile was determined.

## 2. Materials and Methods

### 2.1. Materials

The cross-linked β-cyclodextrin with polyethylene glycol diglycidyl ether (βCPCD) was synthetized at Cranfield University in the U.K., as previously described [17,18]. The soluble (2-hydroxy-3-*N*,*N*,*N*-trimethylamino)propyl-beta-cyclodextrin polymer (QAβCDP), soluble beta-cyclodextrin polymer cross-linked with epichlorohydrin (βCDP) were purchased from Cyclolab Ltd. (Budapest, Hungary). Curcumin, porcine gastric mucin (type II, Mw~640 kDa) and other reagents were obtained from Sigma-Aldrich Ltd. (Budapest, Hungary).

### 2.2. Phase-Solubility Test

The phase-solubility test was performed by adding a fixed excess amount of curcumin powder to 2.0 mL aqueous solutions containing two different types of cyclodextrin polymers, βCPCD and βCDP, at increasing concentrations (0.5–10.0 *m*/*m* %). In the capped vials, excess amounts of curcumin powder (20.0 mg) were measured, in which a constant volume of purified water and increasing concentrations of each cyclodextrin were placed. The vials were vortexed for 30 s to achieve well-mixed dispersions. They were rotated at room temperature at 50 rpm and protected from light. After 24 h, each vial was centrifuged at 4500 rpm for 20 min. The samples were taken from the clear supernatant, and the curcumin content of the samples was analyzed by UV spectrophotometer (Shimadzu UV-1900) at 430 nm. The phase-solubility profiles of curcumin were achieved by plotting the solubility of curcumin versus the *m*/*m* % concentration of the cyclodextrin polymers. The API loading capacity was calculated by Equation (1).

(1) $$\mathrm{API}\;\mathrm{loading}\;\mathrm{capacity}\;\left(‰\right)=\frac{\mathrm{Weight}\;\mathrm{of}\;\mathrm{dissolved}\;\mathrm{API}}{\mathrm{Weight}\;\mathrm{of}\;\mathrm{final}\;\mathrm{product}}\times 1000$$

The weight of the final product was calculated from the measured mass of polymer and the dissolved curcumin determined by UV spectrophotometry.

### 2.3. Preparation of Complexes

Using a 10.0 *m*/*m* % polymer solution in ultrapure water prepared by Millipore Direct-Q 5UV system (Merck Millipore, Burlington, MA, USA), an excess amount of curcumin was dispersed, then stirred at 50 rpm for 24 h to reach equilibrium. The suspension was centrifuged at 4500 rpm for 20 min. The supernatant was frozen at −110 °C, and the sample was lyophilized using a ScanVac CoolSafe freeze dryer (Labogene, Allerød, Denmark). The complexes were stored at −20 °C until used in further experiments.

### 2.4. Dynamic Light Scattering (DLS) Measurements of Complexes

The effect of complexation on the average particle size and particle size distribution of 10 *m*/*m* % solution of complexes was measured by DSL. The cyclodextrin polymer solutions and complex solutions were measured using a Malvern Nano-ZS Zetasizer (Malvern Instruments, Malvern, UK) in purified water.

### 2.5. Solid Phase Solubilization Study

An excess of curcumin (5.0 mg) was added to a 10% *m*/*m* % solution of polymer in water (3.0 mL). After stirring for 24 h, the supernatant was removed, filtered and the dissolved curcumin content of the clear filtered supernatant was determined by UV-VIS spectrophotometer (Shimadzu UV-1900) at 430 nm. Then, 1.5 mL of fresh water was added to the remaining solid phase and then stirred for 24 h once more. The supernatant was removed again, filtered and the curcumin content of the supernatant was measured by UV-VIS spectrophotometer. The process was repeated once more to wash twice the reaming solid phase.

### 2.6. Nuclear Magnetic Resonance (^1^H and NOESY NMR) Measurements

When the βCPCD polymer was used, ^1^H NMR measurements were performed on a Bruker Ascend 400 MHz spectrometer with a BBFO probe to examine the solid phase after the washing process. When curcumin was mixed with the polymer in deuterated water (D_2_O) as previously described in Section 2.4, a well-dispersed suspension was observed. The suspension was filtered using cotton wool and the solid was dried under high vacuum and re-dissolved in deuterated dimethyl sulfoxide (DMSO-d_6_) and the spectrum was recorded at ambient temperature for 16 scans. Signals representing the solvents served as internal standards. The solvent peaks were referenced at 2.5 ppm (DMSO-d_6_) and 4.7 ppm (HDO, H_2_O). Peak multiplicities were described as follows: singlet (s), multiplet (m) and broad (br).

### 2.7. Cytotoxicity Test

The cytotoxic effects of the cyclodextrin polymers were evaluated using the MTT test. The Caco-2 cell line was obtained from the European Collection of Authenticated Cell Cultures (ECACC, U.K.). Cells were maintained in Dulbecco’s modified Eagle’s medium (DMEM, Sigma-Aldrich Ltd., Budapest, Hungary) supplemented with 10.0% heat-inactivated fetal-bovine serum (Sigma-Aldrich Ltd., Budapest, Hungary), 1.0% non-essential amino acid (Sigma-Aldrich Ltd., Budapest, Hungary) and penicillin–streptomycin solution at 37 °C in an incubator containing 5% CO_2_. The passage number of the cells was between 37–52. The Caco-2 cell line is a colon epithelial cell line, in which the cells grow tightly together, forming a single cell layer. A total of 10,000 cells/well were seeded on 96-well plates. After 3 days, the medium was removed, and the cells were incubated for 30 min with the solutions of cyclodextrin polymers at 37 °C. Then, the samples were removed, and a 0.5 mg/mL MTT solution (3-(4,5-Dimethyl-2-thiazolyl)-2,5-diphenyl-2H-tetrazolium bromide dissolved in phosphate buffered saline) was added to each well. The plates were incubated for 3 h. Then, the MTT solution was removed and 0.1 mL isopropanol–1 M hydrochloride acid (25:1) was added to each well to dissolve the formed formazan crystals. The absorbance of formazan was measured at 570 nm and the background was measured at 690 nm by the Thermo Fisher Multiskan Go (Thermo Fisher, Waltham, MA, USA) microplate reader.

### 2.8. In Vitro Permeability Study

For in vitro permeability studies, Transwell^®^ polycarbonate filters (area: 1.12 cm^2^, pore size: 0.4 µm) were used to grow Caco-2 monolayers. Studies were started after the initial seeding of 250,000 cells/well and the transepithelial electrical resistance (TEER) was monitored regularly by Millicell ERS Voltohmmeter (Millipore, USA). After TEER reached 900 Ωcm^2^, the permeability tests were performed. A total of 500 µL of 6 *m*/*m* % complex solutions were prepared in Hanks’ Balanced Salt Solution (HBSS) and the cell monolayers were also washed with HBSS. The solutions of complexes were put onto the apical surface of cell layers. The samples were taken from the basal sides of the cell layers containing fetal bovine serum (FBS) at certain time points (0.5 h, 1 h and 2 h) and purified from protein by adding acetonitrile to the sample (1:1 ratio) and centrifuged at 12,500 rpm for 15 min.

The samples were analyzed using an HPLC system (Merck-Hitachi ELITE with photodiode array detector). The column was Agilent HC-C18(2) (150 × 4.6 mm) and kept at 40 °C, and the detector was set at 430 nm. The mobile phase was the mixture of acetonitrile and 2% acetic acid solution (4:6) and a 1.0 mL flow rate was used. The analyses were performed with EZChrom Elite softwareTM (Hitachi, Tokyo, Japan), which was also used for collecting and processing data. In total, 10.0 μL standard solution and purified samples were injected.

The apparent permeability coefficient (*P_app_*) of curcumin was calculated using Equation (2):

(2) $$P_{app}=\frac{dQ}{dt}\times \frac{1}{\left(C_{0}\times A\right)}$$

where *P_app_* is the apparent permeability coefficient (cm/s); *dQ*/*dt* is the permeability rate of substances (mol/s); *C*_0_ is the initial concentration of the substances in the upper compartment (mol/mL); and *A* is the surface area of the membrane (cm^2^).

### 2.9. In Vitro Mucoadhesive Test of Polymer

To reveal the dominant interactions between mucin and the cyclodextrin polymers, dynamic light scattering (DLS) and zeta potential measurements were performed. The QAβCDP (positive control), βCPCD and βCDP were dissolved in pH 1.2 buffer at 10.0 *m*/*m* % concentration. The mucin dispersion was prepared by adding mucin to deionized water in a concentration of 1 g L^−1^. The dispersion was stirred overnight, and finally centrifuged for 5 min at 4500 rpm. The clear supernatant was used in further experiments. The 1:1 ratio of cyclodextrin polymer solutions with water or mucin solution were prepared and the size distribution, zeta potential and mobility were measured using a Malvern Nano-ZS Zetasizer (Malvern Instruments, Malvern, UK).

### 2.10. MicroCT Analysis

A 10.0 *m*/*m* % solution of the polymer complexes was filled directly into 00 size hard gelatin capsules, which were frozen at −110 °C and lyophilized as described above. The formulations were kept at −20 °C until the study. The following microCT protocol was used to determine the formed structures. Measurements were performed with the SkyScan 1272 desktop micro-CT device with the following settings: image pixel size = 5 microns; matrix size = 1344 × 2016 (rows × columns); 50 kV; 200 µA.

### 2.11. Dissolution Test

During the experiment, three parallel measurements were performed with the 00 size capsules prepared as described above. Each capsule contained 50 mg complexes. A total of 300 mL of hydrochloric acid media, pH = 1.2, without pepsin was selected for the dissolution tests. The rotating paddle method with the rotation speed of 75 rpm and 37 °C was set up in a dissolution tester (Erweka DT 800). Samples of 1.0 mL were withdrawn after 5 min, 15 min, 30 min and 1 h. The released amount of curcumin was determined by the HPLC method described above.

### 2.12. Statistical Analysis

For statistical analysis, SigmaStat (version 3.1; SPSS Inc.) and GraphPad Prism 6 software (GraphPad Software Inc., San Diego, CA, USA) were used. Data are presented as means ± SD. Comparisons of two groups were performed by unpaired t-tests, while comparisons of more than two groups were performed using ANOVA. Differences were considered significant at *p* < 0.05.

## 3. Results

### 3.1. Phase-Solubility Test

**Figure 1:**
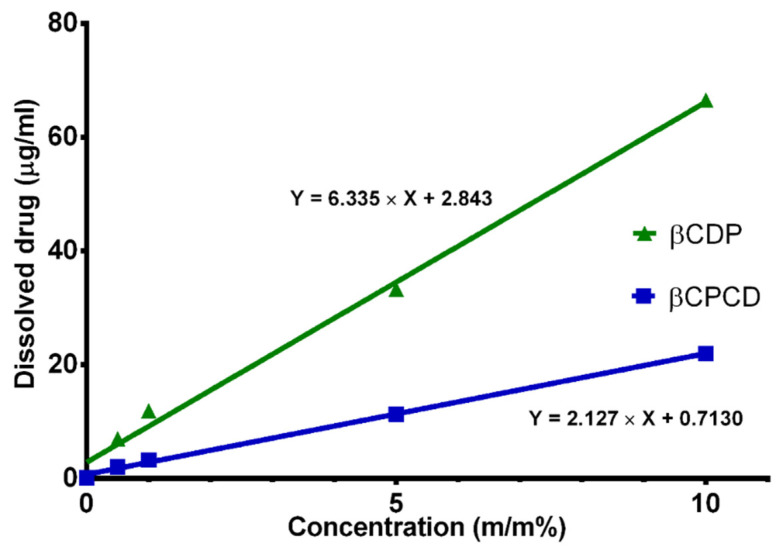
Phase-solubility curve presents the solubility of curcumin (ug/mL) in water versus the *m*/*m* % concentration of the cyclodextrin polymers, βCDP (green curve) and βCPCD (blue curve). Data are presented as means ± SD; *n* = 3.

Figure 1 shows the phase-solubility curve of curcumin in the presence of increasing concentrations of βCDP and βCPCD. Both cyclodextrin polymers were able to improve the water solubility of curcumin in a cyclodextrin concentration-dependent manner.

The capacity results are shown on Table 1. In solution, the βCDP has higher curcumin loading capacities than βCPCD.

### 3.2. Dynamic Light Scattering of Complexes

The average sizes of polymers and the created complexes in purified water were measured by Malvern Nano-ZS Zetasizer and are presented on Table 2. The curcumin–βCDP complex showed smaller size than only βCDP polymer, while the curcumin–βCPCD complex has almost ten times higher particles than the size of the starting polymer.

### 3.3. Solid Phase Solubilization Study

**Figure 2:**
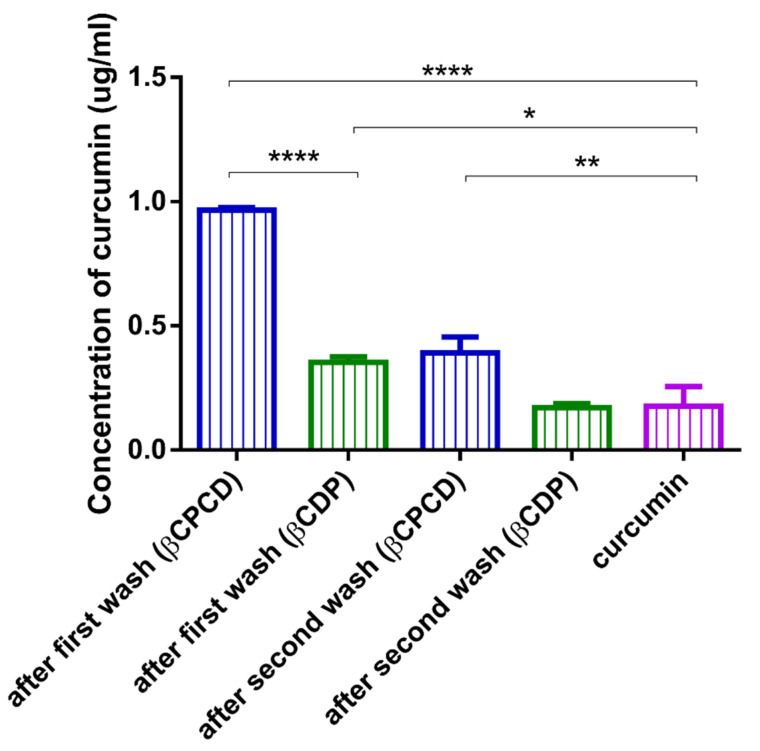
After incubation of excess amounts of curcumin with 10.0 *m*/*m* % βCDP and βCPCD solutions for 24 h, the supernatant was removed and the solid sedimented phase was further washed and incubated with purified water in two washing steps for 24 h in each step. The solubilized curcumin in the supernatant after each wash was determined and compared to the curcumin dissolved without cyclodextrin polymers (data are presented as means ± SD, *n* = 3. Labels of the significant differences are the following: **** *p* < 0.0001, ** *p* < 0.01, * *p* < 0.05).

As there was a significant difference in curcumin solubilization between the two polymers in the phase-solubility test with bigger aggregates observed by DLS after complexation with βCPCD, the solid, sedimented phase of the samples was also examined. The supernatant, containing the soluble complexes, was removed and the solid phase was washed twice with water for 24 h. After the first wash, a significantly higher amount of solubilized curcumin was washed out from the solid phase formed with βCPCD (Figure 2, left, blue box) than βCDP (Figure 2, second from left, green box). After the second wash, the solid phase of the βCPCD sample still contained solubilized curcumin (Figure 2, center blue box), while in the case of the βCDP sample, the curcumin concentration in the supernatant was identical (Figure 2, second from right, green box) to the curcumin own solubility (Figure 2, right, purple box). As expected, the results confirmed that the solubility of the βCPCD polymer in water was affected by the content of curcumin complexed by the network.

### 3.4. NMR Study

**Figure 3:**
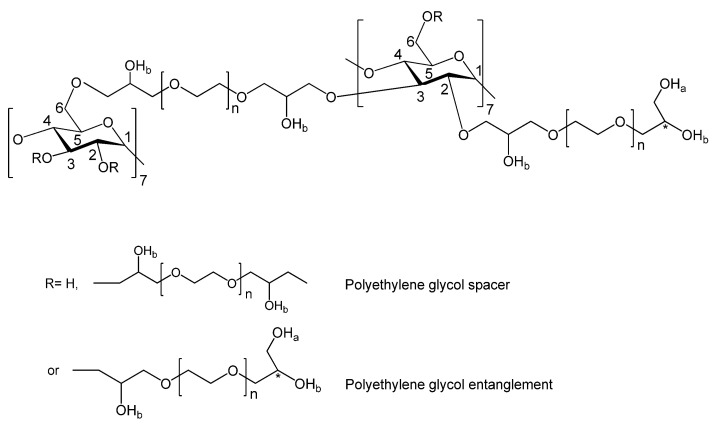
Proposed chemical structure of βCPCD polymer [16].

To further investigate the composition of the solid sedimented phase of the βCPCD– curcumin complex discussed above, the ^1^H NMR spectra were examined and compared with those of the starting materials registered in D_2_O and DMSO-d_6_ (SI, Figure 1 and Figure 2); no reference was used in the deuterated solutions as for the inclusion properties of the βCPCD sample. Figure 3 shows the chemical structure of βCPCD, which was soluble in both solvents, while curcumin floated in D_2_O and totally dissolved in DMSO-d_6_ (Supplementary Figures S1 and S2).

**Figure 4:**
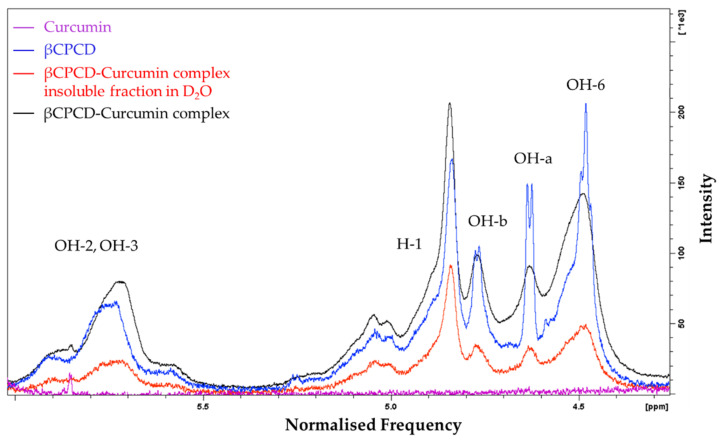
Comparison of ^1^H NMR spectra in DMSO-d_6_ of curcumin (purple), βCPCD (blue), insoluble fraction in D_2_O of βCPCD–curcumin mixture (red) and βCPCD–curcumin mixture (black) at 6.00–4.30 ppm**.**

**Figure 5:**
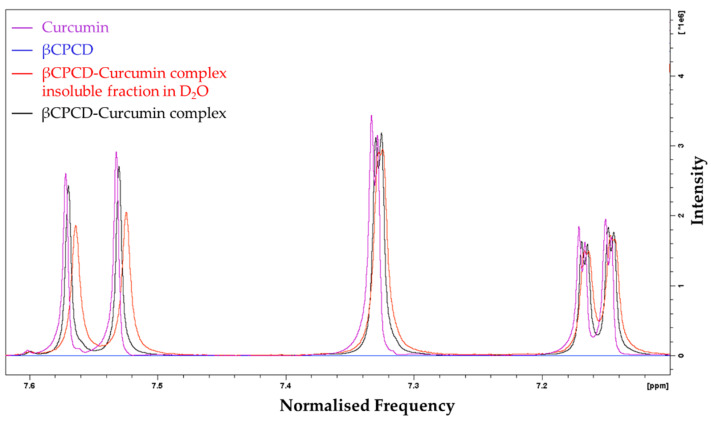
Comparison of ^1^H NMR spectra, in DMSO-d_6_ of curcumin (purple), βCPCD (blue), insoluble fraction in D_2_O of βCPCD–curcumin mixture (red) and βCPCD–curcumin mixture (black) at 7.60–7.10 ppm.

**Figure 6:**
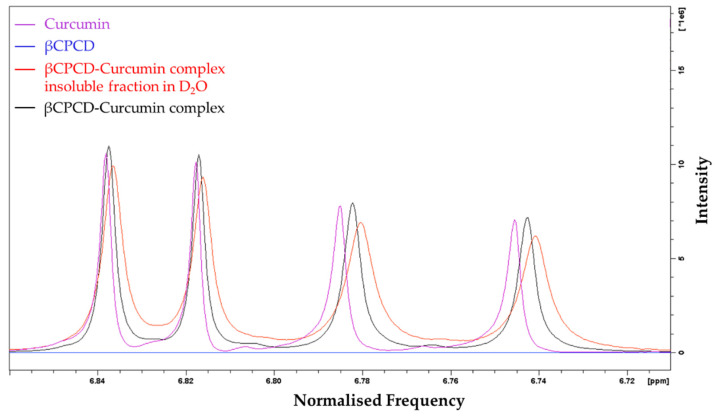
Comparison of ^1^H NMR spectra, in DMSO-d_6_ of curcumin (purple), βCPCD (blue), insoluble fraction in D_2_O of βCPCD–curcumin mixture (red) and βCPCD–curcumin mixture (black) at 6.86–6.71 ppm**.**

When curcumin was mixed with the polymer (as in Section 2.2) in D_2_O at room temperature, for 24 h, a well-dispersed yellow-orange suspension was observed to confirm its solubilization in water due to partial complexation in the cross-linked polymeric structure. After filtration of the suspension, the spectrum in DMSO-d_6_ of the insoluble βCPCD–curcumin fraction in D_2_O was compared with the spectrum of the mixture in DMSO-d_6_ and those of the starting materials. As expected, no changes were observed in the number of peaks associated to the two components, confirming that no chemical modification occurred upon their mixing in DMSO-d_6_. We note that (i) the solubility of the βCPCD polymer after complexation decreased in water; (ii) broader peaks were associated to the hydroxyl groups of the βCPCD in the regions of 6.00–4.30 ppm (Figure 4, purple line—curcumin, blue line—βCPCD, red line—βCPCD–curcumin complex fraction insoluble in D_2_O, black line—βCPCD–curcumin complex) were observed, indicating the complexation of the curcumin in the βCPCD network (Figure 4, OH-a and OH-b) and CD toroid (Figure 4, H-1, OH-6); and (iii) a small chemical shift of aromatic (7.60 ppm to 7.10 ppm) (Figure 5) and vinylic protons (6.86 ppm to 6.71 ppm) of curcumin (Figure 6) confirmed the interaction of the two molecules.

**Figure 7:**
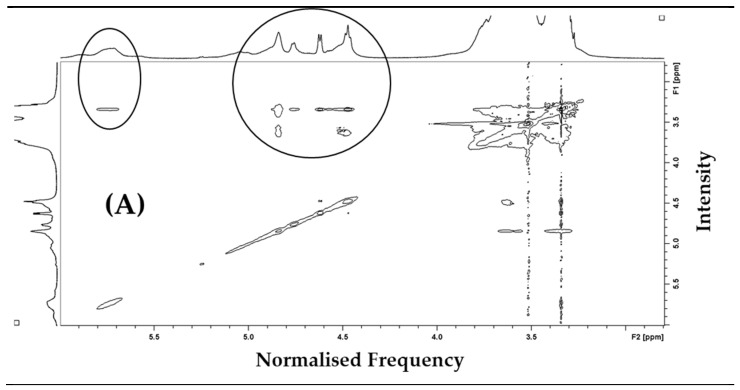

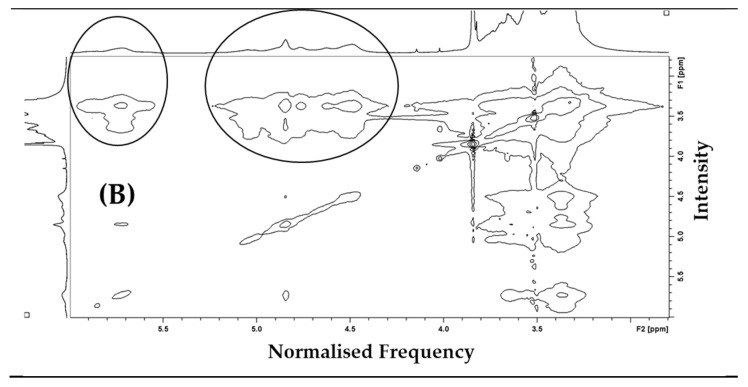
Comparison of 2D NMR NOESY spectra of βCPCD (**A**) and βCPCD–curcumin mixture (**B**) in DMSO-d_6_ at 6.00–2.80 ppm**.**

Additionally, preliminary two-dimensional (2D) NOESY NMR analysis of the βCPCD–curcumin complex in DMSO-d_6_ provided further evidence of the spatial relationships among the protons of βCPCD within the complex when the curcumin guest molecule is placed inside the 3D structure of the cross-linked polymer. Figure 7 reports the 2D NMR NOESY spectra of the βCPCD and βCPCD–curcumin complex in DMSO-d_6_. The intramolecular cross peaks centered at 5.70 ppm (Figure 7, H-1 anomeric protons) and 4.80 ppm (Figure 7, OH-a, OH-b and OH-6) indicated that the curcumin takes on the motional properties of the βCPCD while bound. Further characterization of the βCPCD–curcumin complex formation by using two-dimensional NMR spectroscopy and scanning electronic microscopy is under investigation.

### 3.5. Cytotoxicity Test

**Figure 8:**
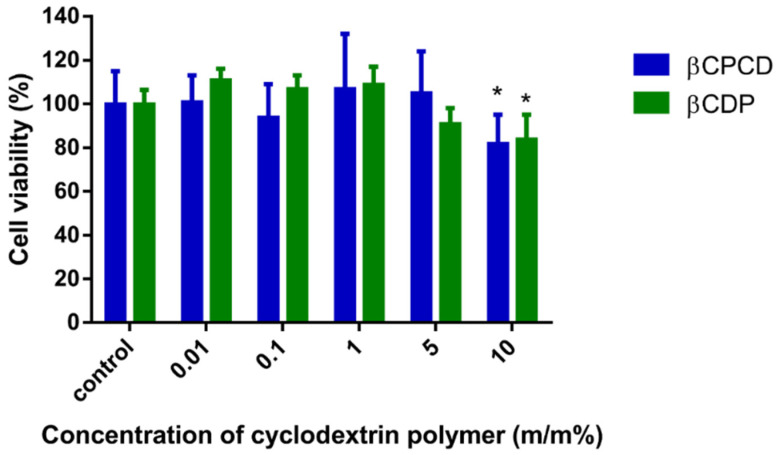
Cytotoxicity of βCDP and βCPCD measured by MTT test on Caco-2 cells after 30 min incubation at 37 °C (data are presented as means ± SD; *n* = 5; * *p* < 0.05 vs. control based on ANOVA).

Figure 8 shows that cyclodextrin polymers were not cytotoxic on Caco-2 cells after 30 min of incubation up to 5.0 *m*/*m* % concentration, but at 10.0 *m*/*m* %, the cell viability decreased significantly compared to the untreated control (*p* < 0.05, *n* = 5).

### 3.6. In Vitro Permeability Study

**Figure 9:**
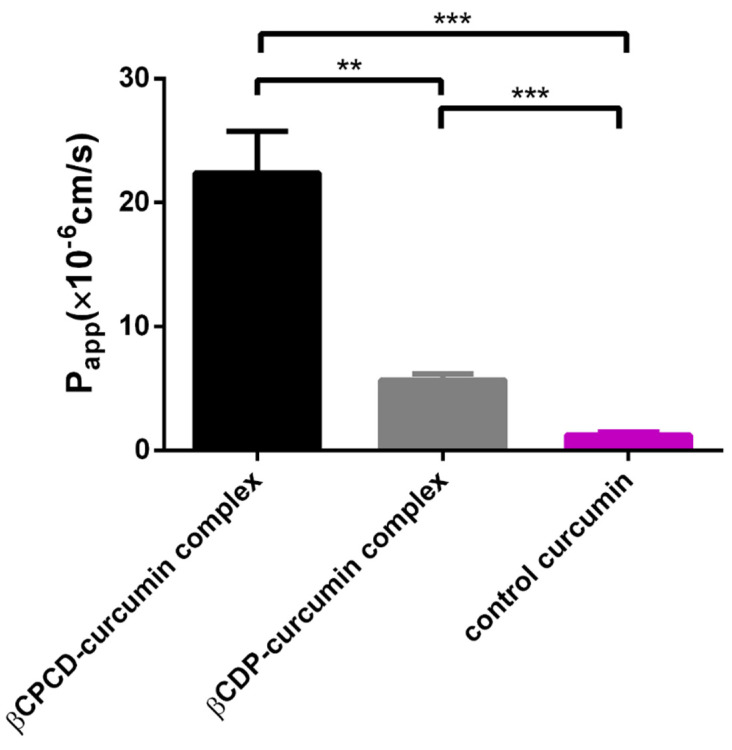
Permeability of curcumin on Caco-2 monolayers treated with curcumin–cyclodextrin complexes. Cyclodextrin polymers significantly increased *P_app_* of curcumin (data are presented as means ± SD, *p* < 0.0005, *n* = 3; *** *p* < 0.0005, ** *p* < 0.01 based on ANOVA) (*P_app_*—apparent permeability coefficient).

The permeability of curcumin using cyclodextrin polymer complexes of 6.0 *m*/*m* % were tested on Caco-2 monolayers. Both polymers were able to significantly improve (*p* < 0.005) the curcumin permeation. The *P_app_* value of curcumin, 1.23 × 10^−6^ cm/s, was increased to 5.70 × 10^−6^ cm/s and 22.40 × 10^−6^ cm/s by the βCDP and βCPCD complexes, respectively (Figure 9).

TEER values of the Caco-2 monolayers were measured before and after the permeability test. No significant decreases in TEER values were observed after the in vitro permeability study, indicating that the integrity of the monolayers was not damaged.

### 3.7. In Vitro Mucoadhesive Test of Polymers

The in vitro mucoadhesive test result is summarized in Table 3. Mucin had an average size of 391.0 ± 7.2 nm with negative zeta potential. A positively charged cyclodextrin polymer, QAβCDP, was used as a positive control, which had positive zeta potential value. In the case of QAβCDP and mucin mixture, we detected increased particle size and increased zeta potential. The βCDP had also a negative charge with a similar size to QABCDP. The size of the βCDP–mucin particles doubled, compared to mucin, while the zeta potential did not increase significantly. The βCPCD polymer showed the highest size increase after the interaction with mucin with the particle diameter of 2052.0 ± 120.9 nm; however, there was no significant change in the zeta potential.

### 3.8. MicroCT Analysis

**Figure 10:**
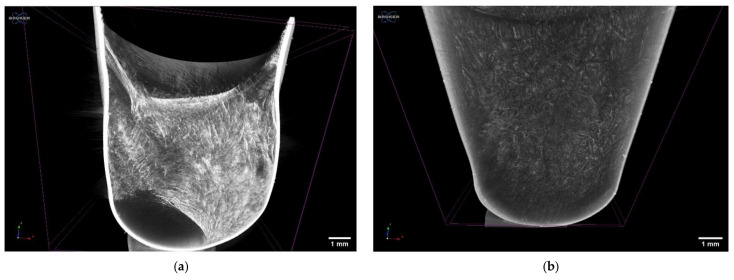
The microCT images of capsules filled with curcumin–βCPCD complex (**a**) and curcumin–βCDP complex (**b**) after lyophilization**.**

The images of microCT scans performed on curcumin–cyclodextrin polymer complexes formulated into hard gelatin capsules are presented in Figure 10. The capsule formulations were prepared by lyophilization, and the complexes showed different structures after removal of the solvent. The cyclodextrin polymer complex formed by βCPCD showed a highly porous structure with filaments (Figure 10a); in contrast, the other complex (curcumin–βCDP complex) created a porous but granular structure with higher density.

### 3.9. Dissolution Test

**Figure 11:**
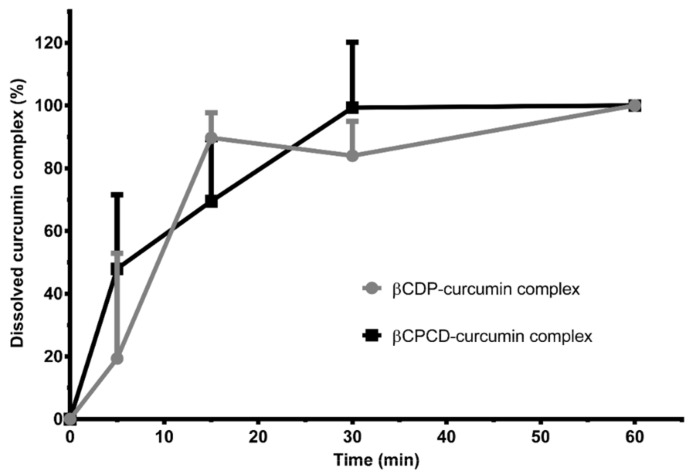
Dissolution curves of hard gelatin capsules filled with curcumin–cyclodextrin polymer complexes (data are presented as means ± SD; *n* = 3).

The capsules filled with the two types of complexes were tested by dissolution tests. During dissolution, complete drug release was observed within 1 h in both cases. In the case of the curcumin–βCDP complex, the total capsule dissolution occurred after 15 min, while in the case of the curcumin–βCPCD complex, the dissolution finished after 30 min. In the case of the curcumin–βCDP complex, complete dissolution occurred after disintegration, while in the second case, the disintegration and the release of the active ingredient into solution took place simultaneously (Figure 11).

## 4. Discussion

A new ‘two in one’ water-soluble β-cyclodextrin polymer (βCPCD) was synthetized recently based on cyclodextrins covalently cross-linked with diepoxides having polyethylene glycol (PEG) spacers [18]. Both polyethylene glycol and β-cyclodextrin are widely used in pharmaceutical formulations, but their covalent bond polymeric combination has not yet been tested for complexation and drug delivery. Curcumin a water-insoluble drug was used as a model drug, to test the complexation properties of water-soluble βCPCD, and compared to those of the water-soluble βCDP. The two polymers differ in their cross-linkers, as βCDP cross-linked with epichlorohydrin; thus, differences in their physical and drug complexing properties were expected. In general, PEG chains can be hydrated, causing larger hydrodynamic radius of the polymer molecules. This was reflected in the DLS results, where βCPCD showed a larger size in two different experiments at two different pH values compared to βCDP. It is especially interesting to associate their molecular weights to the DLS data, as βCPCD is less than 5 kDa calculated from GPC experiments using the PEG/PEO standards, while βCDP is around 105 kDa determined by the static light scattering method referred by the manufacturer. The two methods are not identical, but the dynamic light scattering test results indicate that βCPCD can be hydrated with water at a much higher extent, causing a larger hydrodynamic radius. The other property of βCPCD is the mucoadhesion. The interaction of mucin and the polymers was tested in solution at pH 1.2 by DLS. As a positive control, the positively charged QAβCDP was used. It is well known that mucin has a negative charge, which ensures the attachment of positively charged particles and polymer chains on its surface, and on the other hand, mucin also has the ability to form gel and adhere polymers. In the case of the QAβCD and mucin mixture, we detected interconnected, significantly larger colloidal molecules whose zeta potential was increased compared to mucin. Like mucin, βCDP also had negative charges, but the polymer structure gave an opportunity for association. The formed mucin–βCDP particle size is doubled, while the zeta potential did not increase significantly. The βCPCD polymer was cross-linked with PEG, with presumed mucoadhesive properties. Indeed, the highest size increase was observed with βCPCD, even if the zeta potential showed just a minor change compared to mucin. Interestingly, PEG was reported earlier to be non-mucoadhesive [41], but it can enhance the mucoadhesion of other polymers such as poly(2-hydroxyethyl methacrylate) [42]. Mucins contain numerous hydrogen-bonding active groups and this is the molecular basis of the primary interaction for mucins [43]. It seems that PEG increases the interaction of βCPCD with mucin by hydrogen bonding, causing the enhanced aggregation of βCPCD and mucin compared to βCDP and QAβCD. PEG seems to be more effective to increase mucoadhesion than epichlorohydrin cross-linker, which can contribute to the enhanced drug penetration, but this relationship should be confirmed.

**Figure 12:**
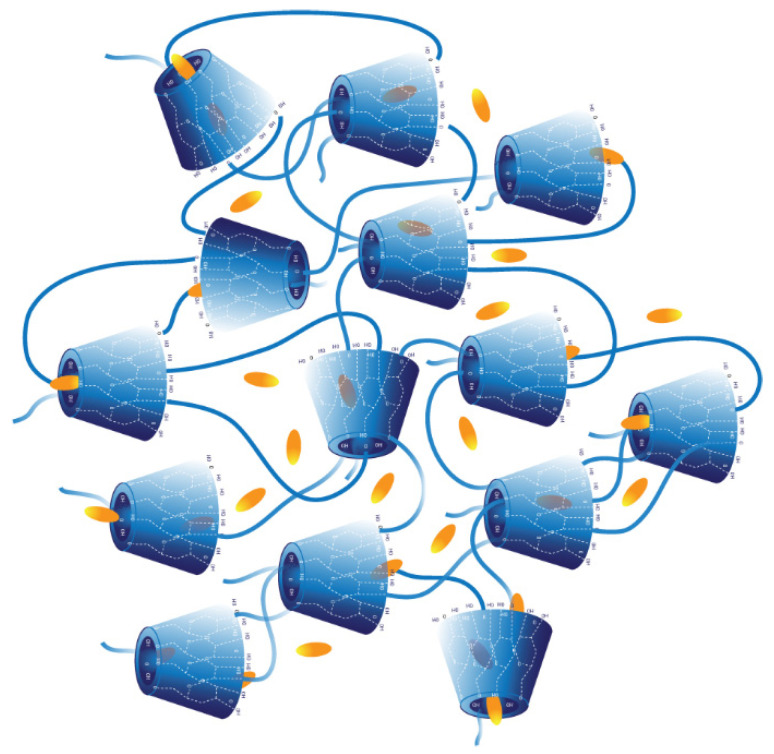
Schematic representation of βCPCD–curcumin complex. The yellow-colored curcumin molecules are entrapped in the β-CD cavity and in the 3D cross-linked area.

Phase-solubility experiments showed that βCDP solubilized curcumin more effectively than βCPCD; however, the size of the complex particles showed an interesting phenomenon. The particle size of the βCPCD–curcumin complex was almost eight times bigger than βCDP. This indicates the formation of an aggregated structure with a possibly lower solubility in water. In general, the complexation ability of the cyclodextrin polymers can be explained by their 3D conformation in water; they have two different regions where the curcumin can be housed, (i) the toroid structure of CD or (ii) the network formed by the cross-linkers in the polymers. However, PEG chains in βCPCD enhance the complexation ability by the formed 3D network with its more flexible structure (Figure 12).

A possible explanation for the solubility differences between the two polymers could be that due to the precipitation of the βCPCD–curcumin complex into the sedimented solid phase, the curcumin concentration in the supernatant solution decreased. To confirm this hypothesis, the solid phase was washed, and we found that the βCPCD–curcumin complex could be further dissolved from the sediment. The presence of the βCPCD–curcumin complex in the sediment was proved by NMR. According to these results, the solubility of both the guest and the host components is affected upon mixing. The curcumin is trapped in the βCPCD polymeric network while the solubility of the polymer is decreased. Interestingly, this could not be concluded from the shape of the phase-solubility diagram, as it was also linear in the case of βCPCD. A possible explanation could be that the applied host molecule was a polymer, which contains molecules with different molecular weights and with different solubility upon complexation. Applying different concentrations in the phase-solubility test, the molecular fraction with less solubility precipitated proportionally, resulting in a linear phase-solubility curve. This hypothesis should be further investigated. Of note, the loading capacity of the polymers was low. This can be partially explained by the way of calculation of the loading capacity of the polymer; due to their high molecular weight, large amounts of polymers should be used to obtain a suitable molar ratio for complexation, which reflects in the smaller value of the loading capacity. The loading capacity could be improved by using other complexation methods and βCPCD kneading could be an alternative method; however, this theory should be confirmed experimentally. Curcumin was used as a model drug in the experiments. Indeed, the formulated dose is small compared to the therapeutically applied doses, but βCPCD increased its permeability twenty times. Due to this positive effect of the polymer on bioavailability of curcumin, the orally applied dose could be reduced. More potent drugs with small doses (< 1mg) would be more suitable to form therapeutically relevant new drug formulations.

In the cytotoxicity test, both polymers showed toxic properties only at higher concentrations (10.0 *m*/*m* %); however, more than 80% of the cells remained viable after 30 min of incubation. TEER measurements were carried out before and after the permeability tests to confirm that cyclodextrin polymer complexes did not induce cell toxicity or the disruption of the monolayer’s integrity during the 2 h of permeability test. TEER values did not decrease significantly after the in vitro permeability studies, showing that the functionality and the integrity of the monolayers were not damaged by cyclodextrin polymers at the applied 6.0 *m*/*m* %. βCPCD improved the curcumin permeability to a significantly higher extent than βCDP, which can be explained by its stronger mucoadhesion and improved contact with the surface of the cell layer. Indeed, the larger hydrodynamic radius creates a larger surface of the complex particles, improving the diffusion of curcumin from the complex into the cell layer. On the other hand, βCPCD can more efficiently deliver the complexed curcumin to the cell membrane, and possibly, the hydrated PEG chains may easier pass through the unstirred water layer on the surface of the cell layer.

Finally, the structure analysis of the formed capsules demonstrated that βCPCD forms a filament-like structure after lyophilization, which swelled during dissolution due to the PEG cross-linker. Nevertheless, the dissolution of both formulations was fast—within 1 h, complete curcumin release was observed.

## 5. Conclusions

In conclusion, the new ‘two in one’ PEG–βCD polymer was applied for the first time successfully to the complexation and formulation of curcumin. The PEG chains provide the cyclodextrin-based polymer with some beneficial properties, including mucoadhesion and complexation. Both PEG and βCD are widely applied pharmaceutical excipients, and thus, the new PEG–βCD system formed through covalent cross-linking could be considered as a new alternative for the formulation of matrix systems for poorly soluble drugs.

## Figures and Tables

**Table 1:** Curcumin loading capacities of βCDP and βCPCD in water (data are presented as means ± SD; *n* = 3).

Complex	Capacity ‰ ± SD
βCDP	0.332 ± 0.004
βCPCD	0.220 ± 0.007

**Table 2:** The average sizes of βCDP and βCPCD and their curcumin complexes in purified water (data are presented as means ± SD; *n* = 5).

Type of CD Polymer	Size of CD Polymer ± SD (nm)	Size of Curcumin Complex ± SD (nm)
βCDP	21.1	18.4
βCPCD	255.0	1978.0

**Table 3:** Interaction of mucin with different cyclodextrin polymers measured by DLS and zeta potential (data are presented as means ± SD, *n* = 5).

Sample Name	Size ± SD (nm)	Zeta Potential ± SD (mV)	Mobility ± SD (µm cm/s)
Mucin	391.0 ± 7.2	−3.12 ± 0.36	−0.2445 ± 0.0276
QAβCDP	9.3 ± 0.2	2.28 ± 0.65	0.1785 ± 0.0508
QAβCDP + Mucin	1326.0 ± 89.7	−0.77 ± 0.65	−0.0603 ± 0.0976
βCDP	11.9 ± 0.2	−2.02 ± 0.16	−0.1584 ± 0.0126
βCDP + Mucin	803.0 ± 22.0	−2.34 ± 0.22	−0.1835 ± 0.0170
βCPCD	33.1 ± 0.2	0.08 ± 7.07 × 10^−5^	0.006 ± 7.7800 ×10^−6^
βCPCD + Mucin	2052.0 ± 120.9	−2.40 ± 0.27	−0.1877 ± 0.0213

## Data Availability

Not applicable.

## Supplementary Materials

The following are available online at https://www.mdpi.com/article/10.3390/pharmaceutics13101710/s1. Figure S1, ^1^H NMR spectra of βCPCD in D_2_O and in DMSO-d_6_; Figure S2, ^1^H NMR spectra of curcumin in D_2_O and in DMSO-d_6_; Figure S3, ^1^H NMR spectra of βCPCD–curcumin mixture in D_2_O and in DMSO-d_6_; Figure S4, Full ^1^H NMR spectra of insoluble in D_2_O βCPCD–curcumin mixture registered in DMSO-d_6_; Figure S5, ^1^H NMR spectrum of βCPCD polymer in DMSO-d_6_ with proposed chemical structure and assignments of protons; Figure S6, Full 2D NMR NOESY spectra in DMSO-d_6_ of βCPCD–curcumin complex at 10.0–2.8 ppm; Figure S7, Concentration vs. time dissolution curves of hard gelatin capsules filled with curcumin–cyclodextrin polymer complexes (data are presented as means ± SD; *n* = 3).
