# Supplementary material for: Preformulation Studies and Bioavailability Enhancement of Curcumin with a ‘Two in One’ PEG-β-Cyclodextrin Polymer
Source: Pharmaceutics. 2021 Oct 16;13(10):1710. doi: 10.3390/pharmaceutics13101710 (PMC8537279; doi:10.3390/pharmaceutics13101710)
Supplement: Supplementary file 1 [file pharmaceutics-13-01710-s001.zip › pharmaceutics-1383376-SM.pdf]

pharmaceutics

MDPI

Supplementary Materials: Preformulation Studies and Bioavailability Enhancement of Curcumin with a 'Two in One' PEG-β-Cyclodextrin Polymer

Ádám Haimhoffer, Eleftheria Dossi, Monika Béresová, Ildikó Bácskay, Judit Váradi, Ashfaq Afsar, Ágnes Rusznyák, Gábor Vasvári and Ferenc Fenyvesi

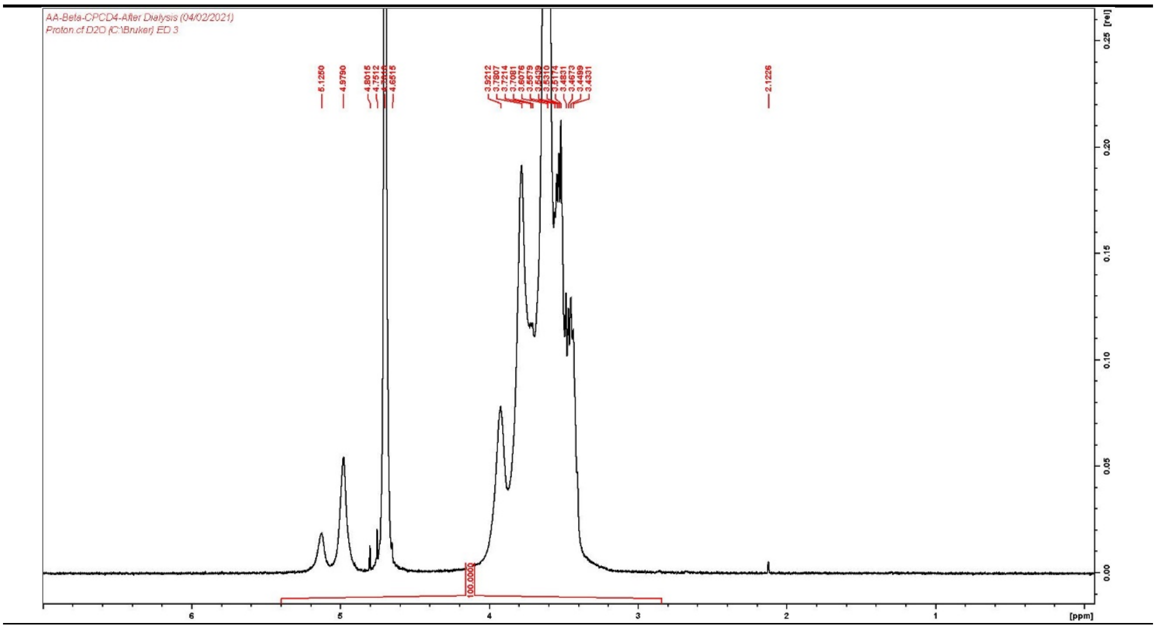

(a)

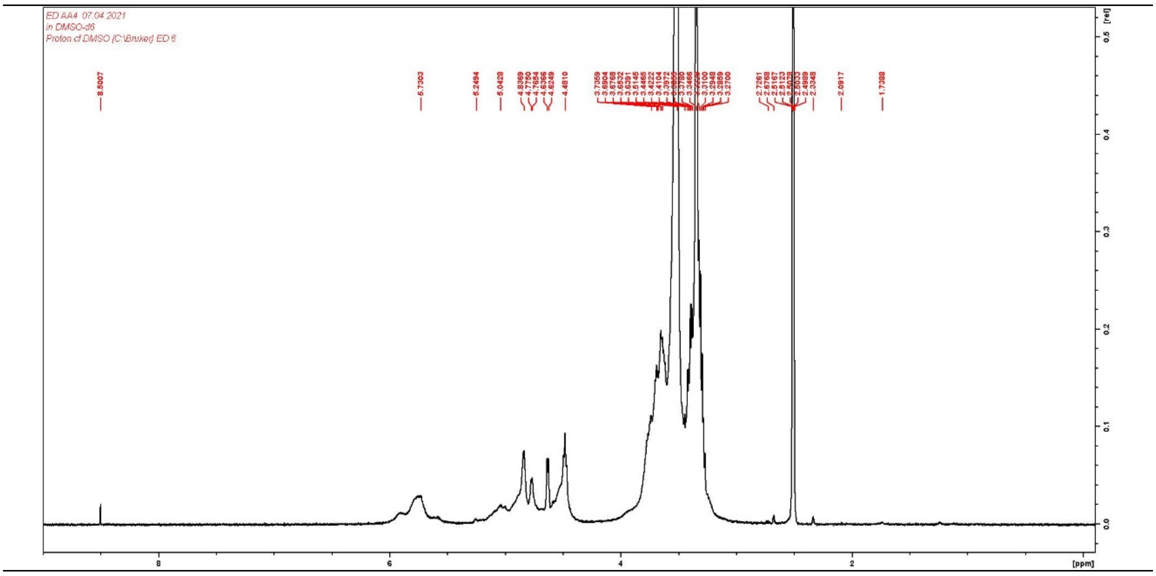

(b)

Figure S1. ^{1}H NMR spectra of βCPCD in D_2O (**a**) and in DMSO-d_6 (**b**).

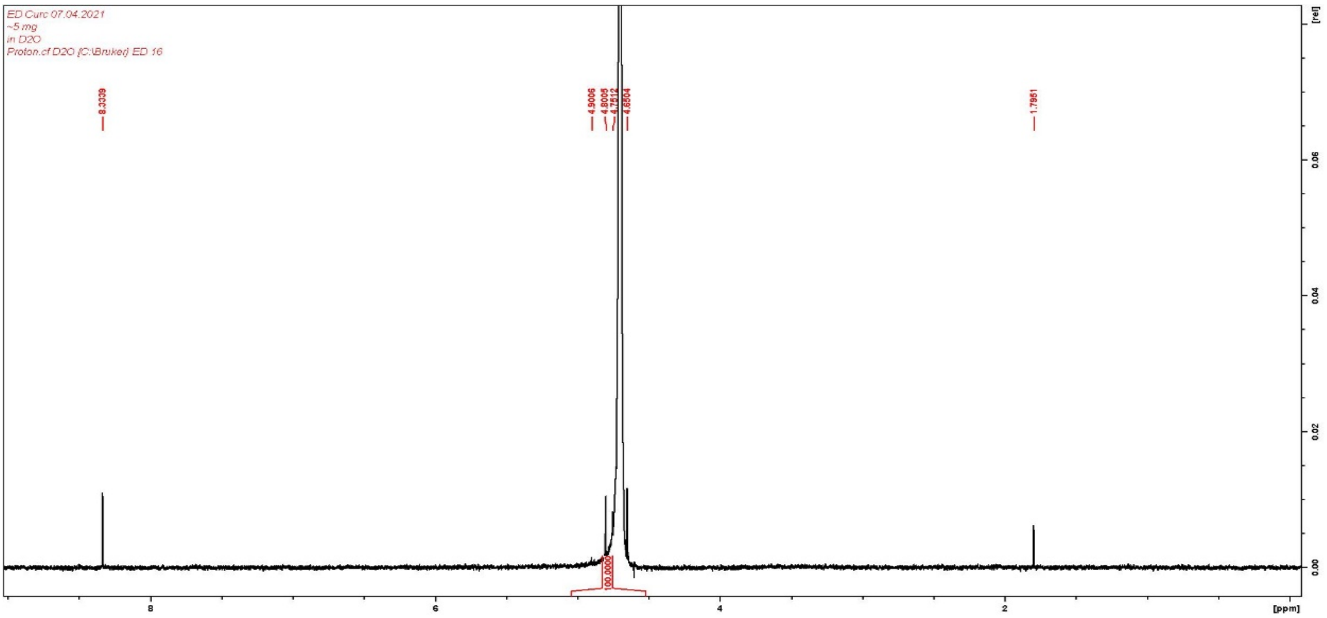

(**a**)

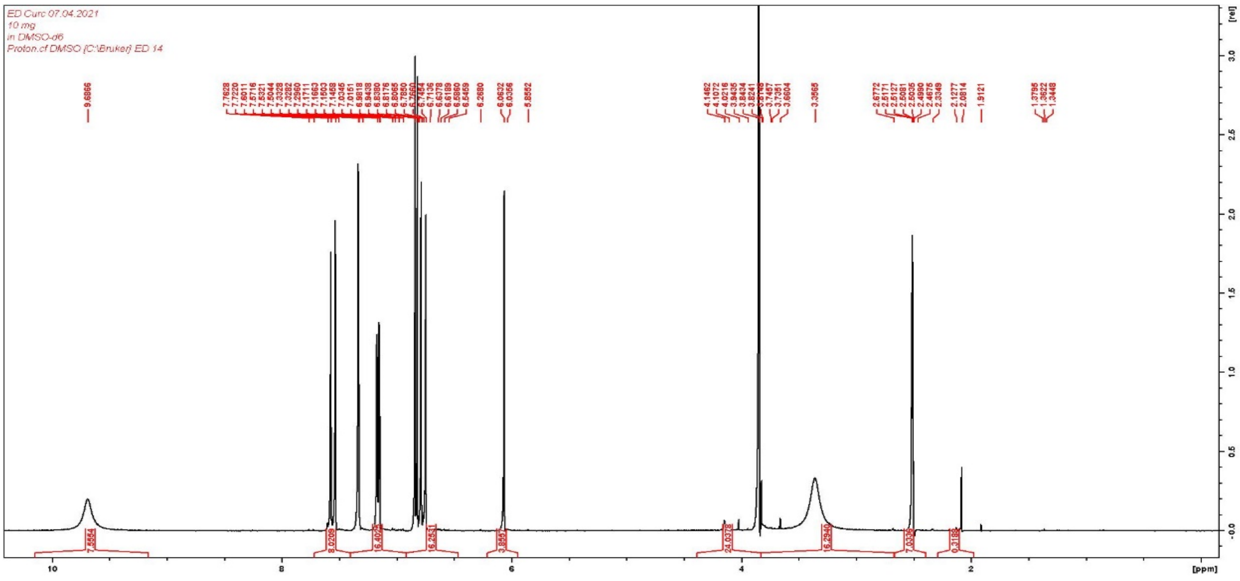

(**b**)

Figure S2. ^{1}H NMR spectra of curcumin in D_2O (**a**) and in DMSO-d_6 (**b**).

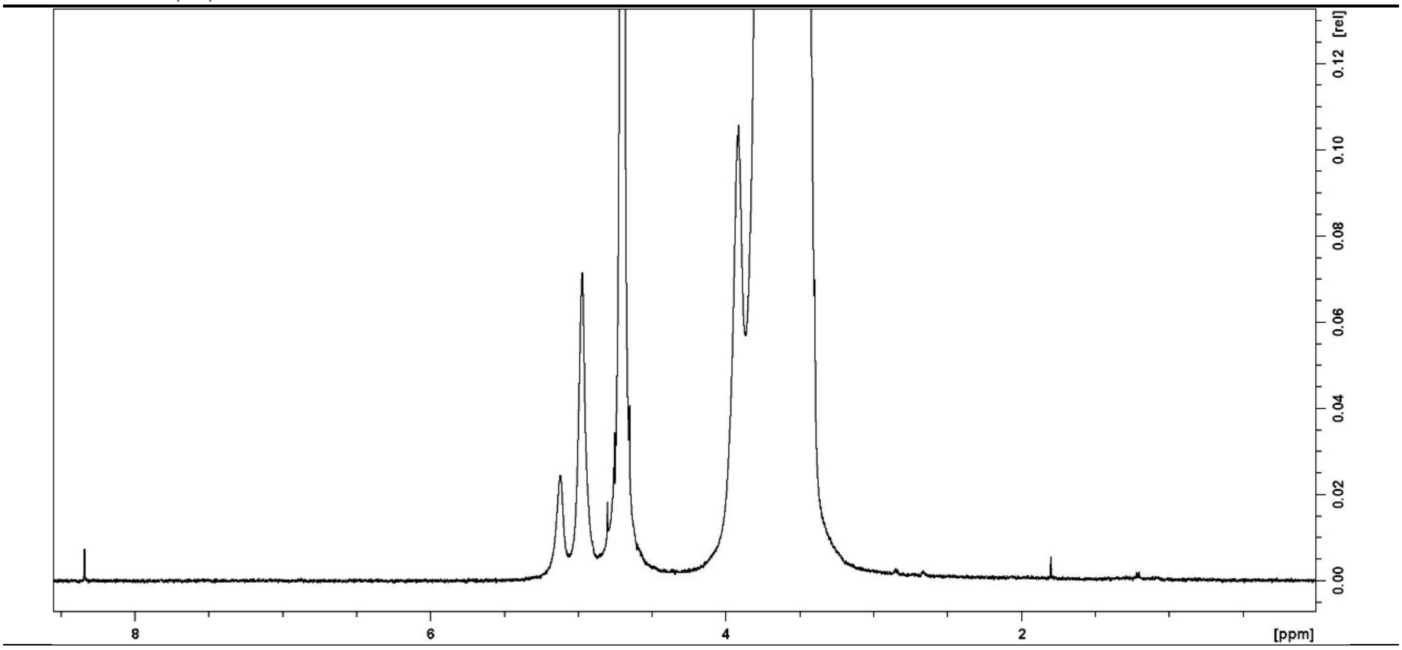

(**a**)

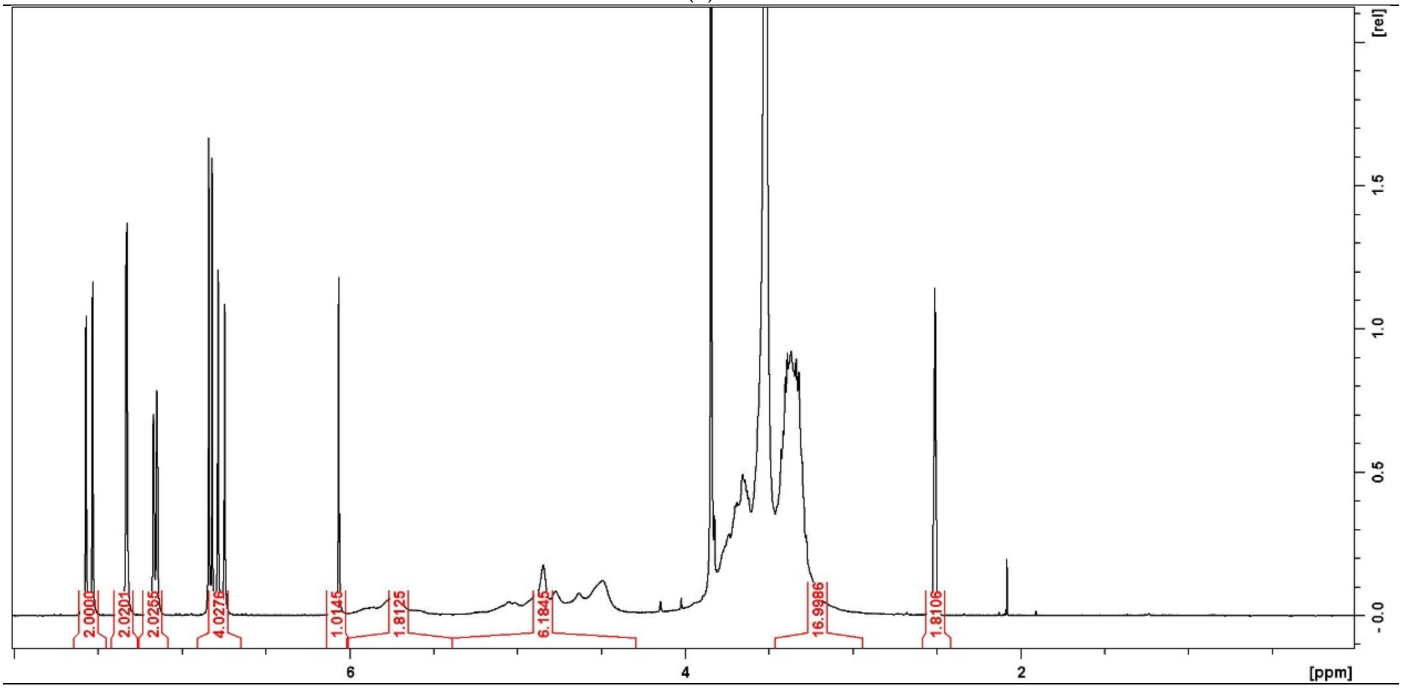

(**b**)

Figure S3. ^{1}H NMR spectra of βCPCD-curcumin mixture in D_2O (**a**) and in DMSO-d_6 (**b**).

The suspension in D_2O was then filtered using cotton wool and the solid was dried at high vacuum and re-dissolved in DMSO-d_6 and the spectrum recorded (Figure S4).

As from the high solubility of the βCPCD in D_2O, the filtrated was expected to be only curcumin but surprisingly was a mixture of the two as from Figure S4. So, the solubility of both components is affected upon mixing. The curcumin (Quantity to be identified) is trapped in the polymeric network while the solubility of the polymer is decreased. We have observed the same behaviour here at Shrivenham, when βCPCD is mixed with inorganic salts such as NaCl and $NaCO_3$.

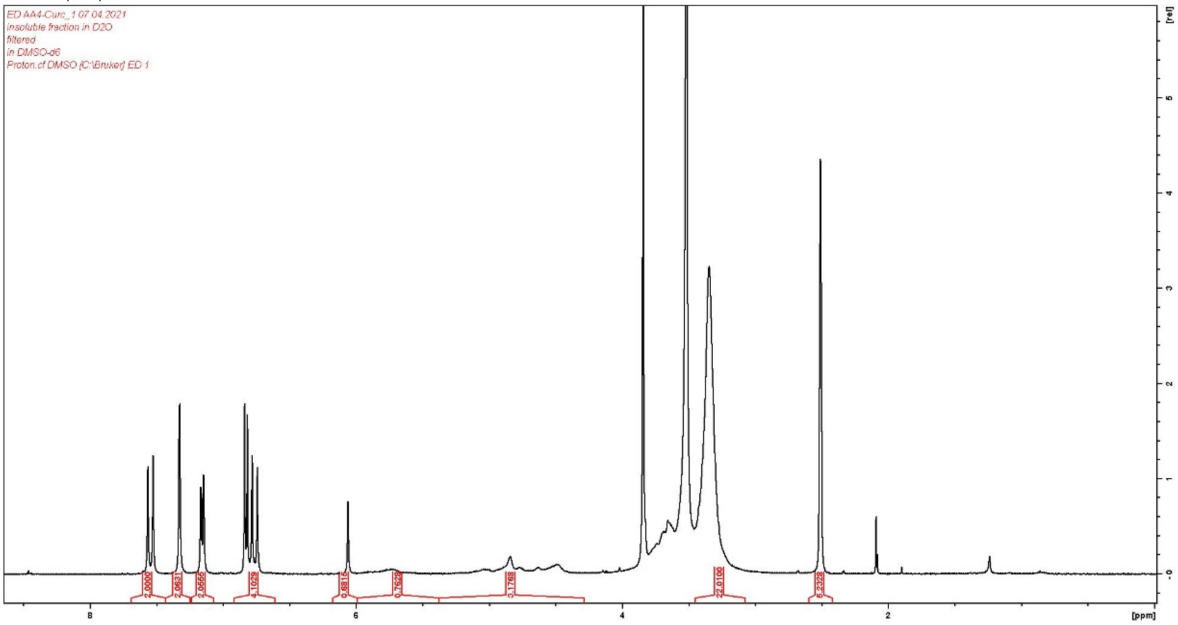

Figure S4. Full ^{1}H NMR spectra of insoluble in D_2O βCPCD-curcumin mixture registered in DMSO-d_6.

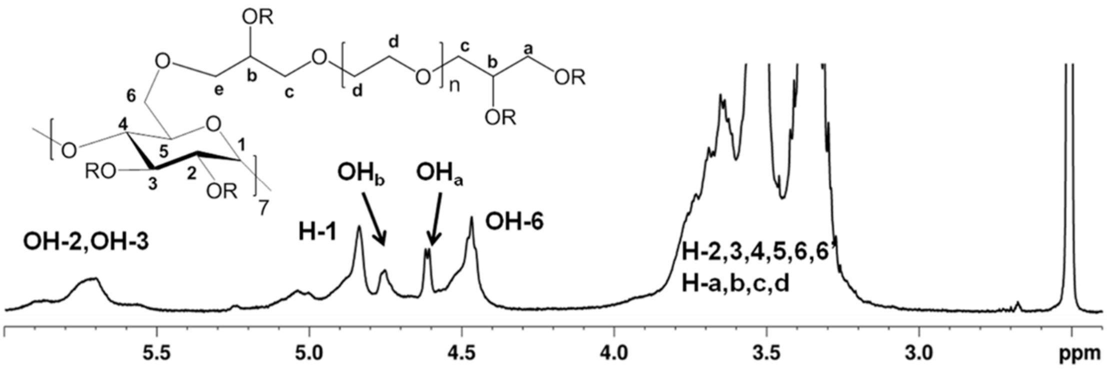

Figure S5. ^{1}H NMR spectrum of βCPCD polymer in DMSO-d_6 with proposed chemical structure and assignments of protons.

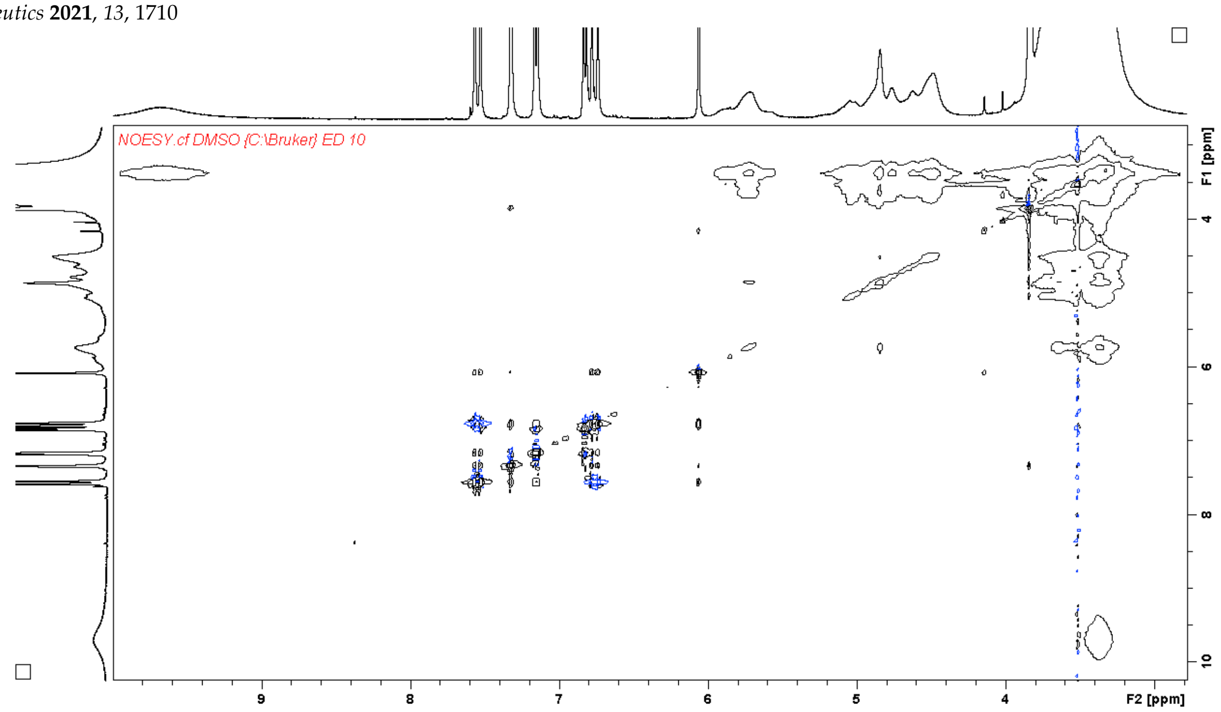

Figure S6. Full 2D NMR NOESY spectra in DMSO-d6 of βCPCD-curcumin complex at 10.0–2.8 ppm.

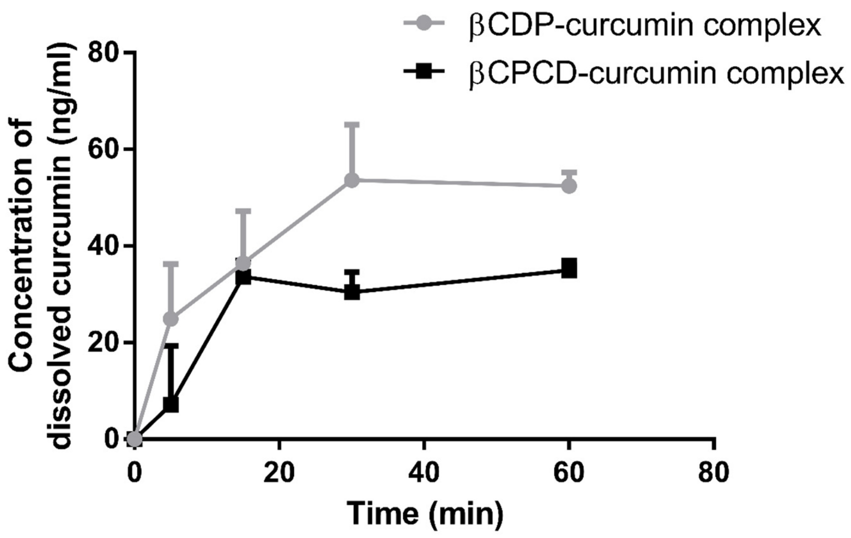

Figure S7. Concentration vs time dissolution curves of hard gelatin capsules filled with curcumin-cyclodextrin polymer complexes (data are presented as means ± SD; *n* = 3).
